# New Epoxy Thermosets Derived from Clove Oil Prepared by Epoxy-Amine Curing

**DOI:** 10.3390/polym12010044

**Published:** 2019-12-27

**Authors:** David Santiago, Dailyn Guzmán, Xavier Ramis, Francesc Ferrando, Àngels Serra

**Affiliations:** 1 Eurecat—Chemical Technology Unit, c/Marcel lí Domingo 2, Edif. N5, 43007 Tarragona, Spain; dailyn.guzman@eurecat.org; 2 Thermodynamics Laboratory, ETSEIB Universitat Politècnica de Catalunya, Av. Diagonal 647, 08028 Barcelona, Spain; ramis@mmt.upc.edu; 3 Department of Mechanical Engineering, Universitat Rovira i Virgili, Av. Països Catalans 26, 43007 Tarragona, Spain; f.ferrando@urv.cat; 4 Department of Analytical and Organic Chemistry, Universitat Rovira i Virgili, c/Marcel·lí Domingo 1, Edif. N4, 43007 Tarragona, Spain; angels.serra@urv.cat

**Keywords:** thermosets, eugenol, renewable resources

## Abstract

New thermosets from a triglycidyl eugenol derivative (3EPOEU) as a renewable epoxy monomer were obtained by an epoxy-amine curing process. A commercially-available Jeffamine^®^ and isophorone diamine, both obtained from renewable resources, were used as crosslinking agents, and the materials obtained were compared with those obtained from a standard diglycidylether of bisphenol A (DGEBA). The evolution of the curing process was studied by differential scanning calorimetry and the materials obtained were characterized by means of calorimetry, thermogravimetry, thermodynamomechanical analysis, stress–strain tests and microindentation. 3EPOEU formulations were slightly less reactive, and the thermosets obtained showed higher *T*_g_s than those prepared from DGEBA, since they had higher crosslinking density than formulations with DGEBA because of the more compact structure and higher functionality of the eugenol derivative. 3EPOEU thermosets showed good thermal stability and mechanical properties. The results obtained in this study allow us to conclude that the triglycidyl derivative of eugenol, 3EPOEU, is a safe and environmentally friendly alternative to DGEBA.

## 1. Introduction

Epoxy thermosets have been extensively used in coatings, adhesives, structural applications or electronics because of their good mechanical properties, relatively low shrinkage and high thermal and chemical resistance [1,2]. One of the most valuable characteristics of these materials is their versatility, which is based not only in the epoxy resin structure, but also in the curing agent selected, both defining the curing conditions and final characteristics of the final thermoset. The final performance of epoxy thermosets can also be improved by the addition of fillers, rubbers, reactive or unreactive diluents and other types of modifiers.

The most common starting compound in the production of epoxy resins is bisphenol A, a petroleum-based chemical, which after reaction with epichlorohydrin leads to the formation of the diglycidylether of bisphenol A (DGEBA) with different epoxy equivalent values. Aromatic compounds like bisphenol A are widely used in polymeric materials since they have a high stability, toughness and good thermal and mechanical properties. However, an excessive exposure to bisphenol A may lead to serious health problems, since it has been recognized to be an endocrine disruptor [3,4]. 

For that reason, many studies have been performed to obtain epoxy thermosets from safer alternatives. Moreover, in the last years, the uncertainty in the future availability of oil and the social tendencies towards sustainable development, have pushed the research in materials science to look for new sustainable and renewable resources to synthesize biobased thermosets [5]. Although the most common natural sources to produce epoxy compounds are vegetable oils [6,7,8] and cardanol [9,10], they usually reach low thermosetting performances. Interest in other sources like lignin [11,12], rosin [13], tannin [14] and carbohydrates [15], has been raising recently. Alternative methods for obtaining sustainable epoxy composites have also been explored in recent years [16,17,18,19].

Taking into account the previous issues, our research group centered its attention in the obtaining of new epoxy thermosets for industrial applications based upon safe and renewable epoxy monomers, which fulfill characteristics similar to or even better than DGEBA.

From the many natural substances existing, eugenol (4-allyl-2-methoxyphenol) is an interesting material for the development of epoxy monomers and other functional materials favorable towards the environment [20]. This compound is an aromatic phenol present in clove oil, the essential oil extracted from the clove plant, but it can also be obtained from lignin, and it is active against a great number of bacteria, both Gram-negative and Gram-positive [21]. Its low cost, antimicrobial properties and different chemical groups (phenol and allyl), that can be further modified, make it very attractive as a starting material for the preparation of epoxy thermosets. The rigid, compact and multifunctional structure of eugenol allows us to obtain materials with excellent thermal and mechanical properties for highly demanding applications [20].

Several studies can be found in the literature about the preparation of epoxy derivatives from eugenol. Wan et al. [22] synthesized an epoxy monomer derived from eugenol with a chemical structure similar to DGEBA. Chen et al. [23] provided a strategy for the preparation of epoxy compounds from the esterification of eugenol. Jiang et al. [24] synthesized a novel biobased bisphenol F and its corresponding epoxy resin through a multi-step procedure derived from eugenol. Liu et al. [25] took advantage of the phenol group for connecting two eugenol moieties by reaction with 1,4-dibromobutane and further epoxidation of allyl groups to get a diepoxy monomer. In addition, a trimer from eugenol was synthesized by reacting it with phosphorous oxychloride followed by further epoxidation [26]. This tripoxy monomer was cured with 4,4’-diaminodiphenylmethane and the thermoset obtained was compared with the one obtained from DGEBA, reaching the eugenol derivative better performances.

Our research group has recently published the synthesis of two new epoxy monomers: a triglycidyl eugenol derivative (3EPOEU) [27] and a tetraglycidyl bis-eugenol derivative (4EPOBEU) [28]. Different thermosets were prepared using thiols of different functionalities as crosslinking agents in the presence of several basic catalysts. These reactions follow a click pattern. One of the great advantages of epoxy-thiol click reactions in the curing of epoxy resins is that the crosslinking agents lead to a more homogeneous network, compared with a homopolymerization process. Although good results were reached in both studies, the curing with thiols is not as well-known as the epoxy-amine process, and thiols have some drawbacks related to their odor and the need to use basic catalysts that generally lead to a fast curing. In contrast, amine compounds have been extensively used as curing agents, and a great number of studies can be found in the literature about epoxy-amine reactions [1,2,29]. The epoxy-amine reaction is considered to follow a click procedure, and therefore homogeneous networks are obtained.

**Scheme 1:**
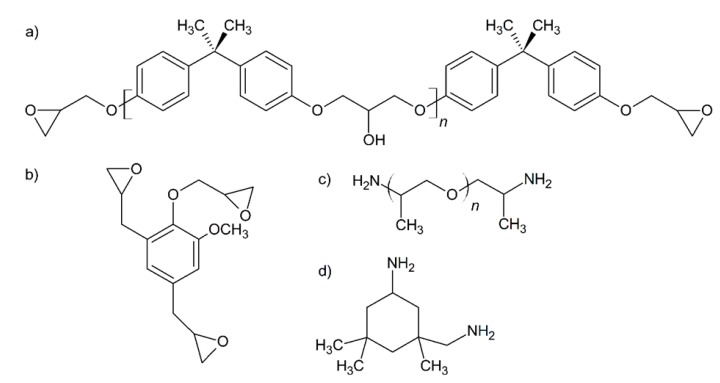
Chemical structures of diglycidylether of bisphenol A (DGEBA) (**a**), triglycidyl eugenol derivative (3EPOEU) (**b**), Jeffamine^®^ D-400 (**c**) and isophorone diamine (IPDA) (**d**).

In the present work, the preparation of new fully renewable epoxy thermosets prepared via epoxy-amine curing has been explored. The trifunctional eugenol epoxy monomer was synthesized according to the procedure described in a previous publication [27] following the concept of green chemistry. Then, the epoxy monomer was crosslinked with two different amine-based compounds as crosslinking agents: isophorone diamine (IPDA), with a rigid structure and derived from a natural source, and Jeffamine^®^ D-400, which is a broadly used aliphatic diamine that introduces flexibility to the network structure. Chemical structures of the starting materials used in the preparation of the thermosets are depicted in Scheme 1. 

Curing process and thermal properties were studied by means of differential scanning calorimetry (DSC) and thermogravimetric analysis (TGA) and the mechanical properties of the materials obtained were investigated by dynamic mechanical analyses (DMA), stress–strain experiments and microindentation. The results obtained were compared with the thermosets prepared from similar formulations but starting from DGEBA. 

## 2. Materials and Methods 

### 2.1. Materials

Eugenol (EU), allyl bromide and oxone (potassium peroxomonosulfate) were purchased from Sigma-Aldrich (Saint Louis, MI, USA) and used without further purification. Diglycidylether of bisphenol A resin (DGEBA, Araldite GY 240) had a weight per epoxy equivalent (EEW) of 182 g/mol and was purchased from Huntsman (Chocolate Bayou, TX, USA). Two different crosslinking agents were used for the preparation of thermosets: polyetheramine Jeffamine^®^ D-400 (JEF) (430 g/mol) with a weight per amine hydrogen equivalent of 115 g/mol, purchased from Huntsmann (Chocolate Bayou, TX, USA) and isophoronediamine (IPDA) (170.3 g/mol), purchased from Acros Organics (Pittsburgh, PA, USA). 

The preparation of 3EPOEU was done according to the method described in a previous work [27], which consists in the allylation of the phenol group, followed by a Claisen rearrangement, a second allylation step of the phenol formed and final epoxidation of the three allyl groups in the formed compound. In the epoxidation step, oxone was used as an oxidizing agent instead of m-chloroperbenzoic acid (MCPBA), following the concept of green chemistry. Other alternatives to MCPBA can be found in the literature such as hydrogen peroxide (H_2_O_2_) [30,31].

### 2.2. Preparation of Curing Mixtures

Four epoxy/amine stoichiometric formulations were prepared with the compositions detailed in Table 1. 3EPOEU formulations were prepared by mixing the compounds dissolved in dichloromethane and then eliminating the solvent at vacuum at room temperature. Formulations with DGEBA (DG) were prepared by mixing the compounds and then homogenizing by manual mixing using a spatula.

### 2.3. Thermal Characterization

The study of the curing was performed by differential scanning calorimetry (DSC) in a Mettler DSC3 + 700/970 (Columbus, OH, USA) calorimeter calibrated using an indium standard (heat flow calibration) and an indium–lead–zinc standard (temperature calibration). A flow of N_2_ at 100 mL/min was used and the weight of the samples for the analysis was 10 mg. The curing process was studied by DSC in the non-isothermal mode at 1, 2, 5, 10 and 20 °C/min from 20 to 275 °C. The glass transition temperatures (*T*_g_s) of the samples once cured were determined in dynamic scans at 20 °C/min from −50 to 120 °C in the case of formulations with Jeffamine^®^ D-400 and from −50 to 200 °C in the case of formulations with IPDA.

The isoconversional activation energy at different degrees of conversion *x* was determined from multiple heating rate experiments using the KAS method [32]:

(1) $$\ln\left(\frac{\beta }{T^{2}}\right)=\ln\left(\frac{A\cdot R}{g\left(x\right)\cdot E}\right)-\frac{E}{R\cdot T}$$

where *β* is the heating rate, *A* is the pre-exponential factor, *E* is the activation energy and *g*(*x*) is an integral function corresponding to the kinetic model. The representation of ln(*β/T*^2^) in front of *1/RT* for the experimental results should produce a straight line, in which the slope of the line is related to the activation energy, *E*, and the ordinate in the origin is related to the pre-exponential factor *A*.

The time needed to reach a given conversion in an isothermal experiment can be determined from the results of the isoconversional analysis of nonisothermal experiments using the following expression:

(2) $$\ln\left(t\right)=\ln\left(\frac{g\left(x\right)}{A}\right)+\frac{E}{R\cdot T}$$

The thermal stability of cured samples was studied by thermogravimetric analysis (TGA), using a Mettler TGA/SDTA 851e thermobalance (Columbus, OH, USA). All experiments were performed under inert atmosphere (N_2_ at 100 mL/min). Pieces of the cured samples with an approximate mass of 8 mg were degraded between 30 and 600 °C at a heating rate of 10 °C/min.

### 2.4. Mechanical Characterization

Dynamic mechanical analyses (DMA) were carried out with a TA Instruments (New Castle, DE, USA) DMA Q800 analyzer. The samples were isothermally cured in a mold at 100 °C for 2 h with a post curing at 200 °C during 2 h in the case of formulations with Jeffamine^®^ D-400 and at 80 °C for 2 h with a post curing at 180 °C during 2 h in the case of formulations with IPDA. The curing schedule was selected after inspection of dynamic curing thermograms by the methodology explained in previous works [33]. 

A three-point bending clamp was used on prismatic rectangular samples (30 mm × 5 mm × 1.5 mm). The apparatus operated dynamically at 3 °C/min from 30 to 120 °C in the case of formulations crosslinked with Jeffamine^®^ D-400 and from 30 to 250 °C with formulation crosslinked with IPDA. The frequency was 1 Hz with and oscillation amplitude 0.1%. Young’s modulus was determined under flexural conditions at room temperature, with the same clamp and geometry samples, applying a force ramp at constant load rate of 1 N/min, from 0.005 to 1.5 N. Stress–strain tests were performed with the film-tension clamp in force-controlled mode. Dog-bone samples were prepared in a mold and tested at a force rate of 1 N/min. 

Microindentation hardness was measured with a Wilson Wolpert 401 MAV (Aachen, Germany) device following the ASTM E384-16 standard procedure. For each material at least 15 determinations were made with a confidence level of 95%. The Vickers hardness number (HV) was calculated from the following equation [34]:

(3) $$HV=\frac{1.8544\cdot F}{d^{2}}$$

where, *F* is the load applied to the indenter in kgf (0.025 kgf) and *d* is the arithmetic mean of the length of the two diagonals of the surface area of the indentation measured after load removal in mm. In all cases, samples were heated at a temperature near *T*_g_ for deleting the thermal history before the experiment.

## 3. Results and Discussion

### 3.1. Study of the Curing Process

**Figure 1:**
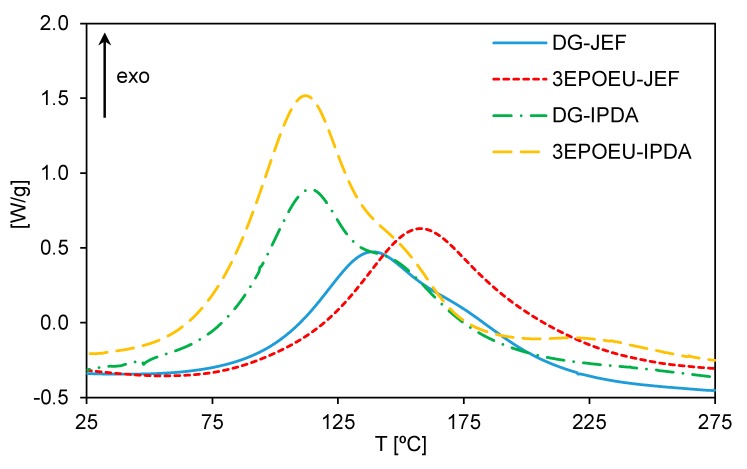
Differential scanning calorimetry (DSC) thermograms corresponding to the dynamic curing at 10 °C/min of the materials studied.

The curing reaction of the 3EPOEU and DGEBA with both amines was studied by calorimetry to know the reactivity of both starting epoxides. The DSC thermograms for all formulations are represented in Figure 1 and Table 2 collects the most interesting data obtained from these experiments. 

From Figure 1, it is clear that IPDA is more reactive than Jeffamine^®^ D-400 for both epoxy compounds, since the exotherms of IPDA mixtures begin at room temperature whereas JEF mixtures curing starts at higher temperatures. Moreover, the shape of the curve is more complex, with a shoulder at high temperatures. The different reactivity of both amine groups of IPDA can be the responsible of the appearance of these shoulders. DSC studies revealed that the reaction of 3EPOEU with JEF begins at slightly higher temperatures than with standard DGEBA, but once started, the height of the peak is higher, indicating a faster curing process. 

When crosslinked with IPDA, the curing reaction begins approximately at the same temperature with both epoxy compounds, but the height of the peak is much higher in the case of the eugenol derivative formulation, which indicates a higher reactivity, related with the lower weight per epoxy equivalent of 3EPOEU.

From the values of Table 2, it can be observed that with both crosslinking agents, the enthalpy of the reaction released is significantly lower in the case of 3EPOEU formulations (around 90 kJ/mol) than the usual for an epoxy-amine reaction, which is described to be around 100–110 kJ/mol ee). The compact structure of the eugenol derivative, with three glycidyl groups linked to an only phenyl ring, could slightly hinder the complete crosslinking due to topological constraints. It can also be argued that topological restrictions could be those responsible for the appearance of a broad exotherm peak around 225 °C in the curing of 3EPOEU-IPDA formulation, because of the high functionality of both compounds and their rigid and compact structure. Whereas the main curve can be attributed to the epoxy-amine reaction, the broad exotherm peak centered in 225 °C could be attributed to the epoxy homopolymerization, which takes place because of the stoichiometric imbalance produced by the hindrance in both compounds [28,35]. It must be noted that the enthalpy of the reaction of the formulation 3EPOEU-IPDA in Table 2 (92 kJ/mol ee) corresponds to both processes (epoxy-amine reaction and homopolymerization). The enthalpy of the reaction of the epoxy-amine process (corresponding to the first peak centered in 130 °C) is around 80 kJ/mol ee (580 kJ/g), which is even less than the value for the formulation 3EPOEU-JEF (88 kJ/mol ee). The topological restrictions occurring in both 3EPOEU and IPDA can lead to this lower enthalpy released for the 3EPOEU-IPDA formulation.

Looking at the glass transition temperatures collected in Table 2, it can be observed how the rigidity, functionality and compactness of the monomers affect this parameter. The thermosets obtained from 3EPOEU showed higher *T*_g_s because they reached a higher crosslinking density than those obtained from DGEBA. Similarly, the use of IPDA instead of JEF also increases the crosslinking density and the rigidity of the materials leading to a notable increase in *T*_g_. The substitution of DGEBA by 3EPOEU and the use of IPDA as crosslinking agent allowed us to reach *T*_g_s of 174 °C, 50 °C higher than the *T*_g_ of the traditional thermoset obtained from DGEBA. In this case, the contribution of homopolymerization, which seems to be more extensive than in DGEBA formulations (Figure 1), is one of the reasons to reach this high value with an aliphatic amine as a curing agent. The use of IPDA as curing agent in the formulation with 3EPOEU allowed us to increase the *T*_g_ values previously reached in 3EPOEU-thiol formulations, with a maximum *T*_g_ of 103 °C for 3EPOEU/hexathiol squalene derivative thermoset [27].

**Figure 2:**
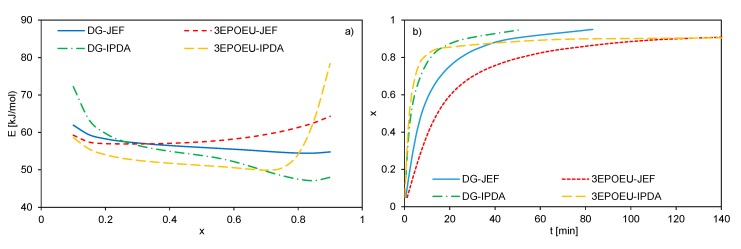
(**a**) Comparison of the activation energy E as a function of conversion *x* during dynamic curing of all formulations. (**b**) Conversion *x* as a function of the simulated reaction time at 120 °C obtained by the isoconversional analysis of nonisothermal experiments (simulations were performed from *x* = 0.05 to 0.95).

Although some reactivity data was obtained from the previous DSC study, the activation energy and the simulated reaction time were calculated according to Equationd (1) and (2) in order to widen the kinetic study. The evolution of the activation energy with the degree of conversion was calculated by the isoconversional method, and the values obtained are shown in Figure 2a. There are no significant differences in the activation energy along the curing process except for formulation 3EPOEU-IPDA, which showed an important increase at the end of the curing. This increase could be attributed tentatively to the homopolymerization process that occurs when curing this formulation (Figure 1). The homopolymerization reaction only occurs at high temperatures because of the high activation energy of this process, which has been reported to be around 80 kJ/mol [36].

The trend of the activation energies for all formulations, due to the compensation effect between the activation energy and the pre-exponential factor [37], does not allow anyone to stablish which one of the curing processes is the most active from the point of view of kinetics. However, the representation of the simulated reaction time at 120 °C, which takes into account both parameters, in front of the conversion achieved, and obtained by the isoconversional analysis of nonisothermal experiments, (Equation (2)) gives a better insight into the evolution of the conversion for each formulation. Figure 2b shows the plot of the conversion x as a function of the simulated reaction time for all formulations. As can be observed, the formulations based on 3EPOEU show slight differences compared with formulations prepared from DGEBA at the early stages of the curing process. However, from x = 0.8, it is very clear that formulations with 3EPOEU take much longer to reach a complete curing than formulations based on DGEBA, which can be related to the high functionality and compact structure of the 3EPOEU molecule. Moreover, the higher reactivity of IPDA as a curing agent in front of the same epoxy monomer is also evidenced.

### 3.2. Thermogravimetric Study

**Figure 3:**
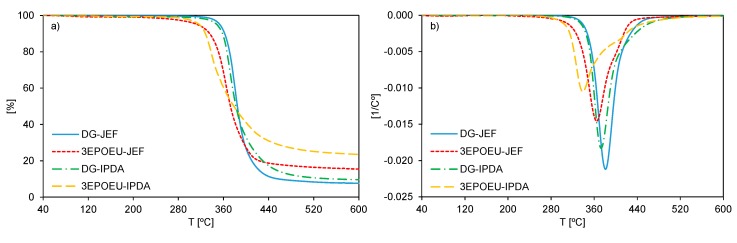
Thermogravimetric analysis (TGA) (**a**) and Differential thermogravimetry DTG (**b**) curves under N_2_ atmosphere at 10 °C/min of the materials studied.

The materials prepared were characterized by thermogravimetry to study their stability at high temperatures. Figure 3a,b show the weight loss curves and the derivatives of weight loss curves against temperature in inert atmosphere. As can be observed, the degradation curves are almost unimodal, with an only shoulder at high temperatures, more visible in the case of the 3EPOEU-IPDA formulation. Table 3 collects the most significant data obtained by this technique. From the values of temperatures of initial weight loss (*T*_5%_) and the temperature of maximum degradation rate (*T*_max_) it can be observed that materials prepared with the tripoxy monomer of eugenol showed slightly lower degradation temperatures than those prepared from DGEBA. However, it can be observed that the degradation rates are significantly slower when using 3EPOEU (Figure 3b) due to its higher crosslinking density that hinders the weight loss. From the values of char residue in the Table 3, it can be observed that the thermosets prepared from eugenol leave a higher charring residue, which could be positive for these materials to get a higher fire retardancy.

The TGA values obtained in the present study also represent an enhancement in the thermal stability in comparison with 3EPO-EU/thiol systems [27]. 3EPOEU/amine thermosets showed slightly higher initial degradation temperatures (around 315 °C) than 3EPOEU/thiol thermosets, which showed degradation temperatures around 300 °C.

### 3.3. Static and Dynamic Mechanical Characterization

**Figure 4:**
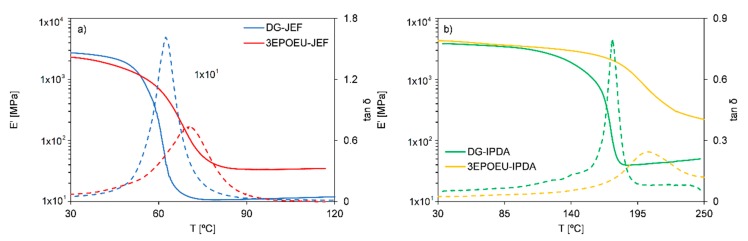
E′ and tanδ curves of the formulations cross-linked with Jeffamine^®^ D-400 (**a**) and isophorone diamine (IPDA) (**b**).

Figure 4 shows the DMA curves of formulations crosslinked with Jeffamine^®^ D-400 (Figure 4a) and IPDA (Figure 4b), and Table 4 collects the most typical thermomechanical data. From the tan δ curves, it can be observed that thermosets derived from eugenol have a broader curve than those prepared from DGEBA, and a higher temperature of the maximum of the peak, which can be attributed to the trifunctionality and to the more compact structure of the 3EPOEU monomer. The thermoset denoted as 3EPOEU-IPDA has a tanδ temperature of 204 °C, which is 30 °C higher than the corresponding material with DGEBA. This value is also much higher than the tanδ temperature obtained in a previous study with 3EPOEU-thiol formulations [27].

A representative parameter of the network homogeneity is the value of width at half height of the peak of the curve (FWHM), which is collected in Table 4. Formulations with 3EPOEU show higher values of FWHM (less homogeneity) than formulations with DGEBA. When cross-linked with IPDA, the FWHM is significantly higher. This result is not surprising considering the compact structure of 3EPOEU molecule (Scheme 1), which leads to a more densely crosslinked network with higher restricted mobility and the occurrence of some homopolymerization reaction.

According to the theory of rubber elasticity, the rubbery modulus *E’*_r_ is proportional to the crosslinking density [38]. Considering the values of *E’*_r_ in Table 4, formulations with 3EPOEU have higher crosslinking density than formulations with DGEBA, mainly because of its tri-functionality and higher amount of crosslinking points by weight unit. A higher crosslinking density leads to a higher *T*_g_ (considered as the maximum of tanδ peak) but it also has to be considered the chemical structure of the network formed. The small and short molecule of 3EPOEU, in contrast to the longer and kinked structure of DGEBA, leads to a higher value of storage modulus in the rubbery region. The homopolymerization of the epoxide, which seems to be quite important in the 3EPOEU-IPDA formulation, contributes to increase the crosslinking density (and, consequently, the relaxed modulus) and the tan δ temperature. Formulations crosslinked with JEF have lower crosslinking density and *T*_g_ than formulations crosslinked with IPDA due to its lower amount of crosslinking points by weight unit.

The mechanical behavior of the thermosets obtained was studied by tensile tests and Table 5 collects the most characteristic values measured at room temperature. Thermosets crosslinked with Jeffamine^®^ D-400 showed approximately the same value of Young’s modulus at room temperature, whereas the materials crosslinked with IPDA show higher values that depend on the structure of the epoxy monomer. The 3EPOEU-IPDA thermoset presents the highest Young’s modulus (about 6 GPa) due to its high crosslinked and compact structure. The 3EPOEU materials studied in this work have higher Young’s modulus than those reported in 3EPOEU-thiol thermosets [27].

Materials obtained from formulations with 3EPOEU show lower stress and strain at break, but higher microindentation hardness than the corresponding materials obtained from DGEBA. Formulations cured with IPDA lead to thermosets with lower strain at break, but with a higher stress at break and higher microindentation hardness than formulation cured by Jeffamine^®^. The plasticizing effect of the long Jeffamine^®^ structure in comparison with IPDA, together with the lower crosslinking density of Jeffamine^®^ formulations, are responsible for this behavior.

Materials with 3EPOEU showed higher stiffness due to 3EPOEU’s higher functionality and to the smaller size of the structural unit between crosslinks than those prepared with DGEBA. This leads to a more compact network, which could restrict its stress–strain capability.

## 4. Conclusions

Different thermosets were prepared from the triglycidyl eugenol derivative with two different amine-based crosslinking agents. The materials obtained were compared with those prepared with a standard DGEBA and were characterized by means of DSC, TGA, DMA, stress-strain tests and microindentation.

The calorimetric study revealed that the curing reaction of conventional and renewable epoxy resins was faster when IPDA is used as a crosslinking agent. The curing of 3EPOEU formulations release lower enthalpy than those with DGEBA, which entails a limited conversion caused by the multifunctionality and compact structure of the eugenol derivative. Homopolymerization of epoxide was detected at high temperatures, especially in the case of our 3EPOEU-IPDA formulation, due to topological constrains. The obtained thermosets with 3EPOEU showed higher glass transition temperatures than the corresponding materials from DGEBA. Materials with 3EPOEU showed degradation temperatures above 300 °C, only slightly lower than those prepared from DGEBA, but slower degradation rates.

3EPOEU thermosets had higher crosslinking density than formulations with DGEBA because of their more compact structure and higher functionality, which led to higher *T*_g_s, but with a more heterogeneous network structure. The small and short molecule of 3EPOEU led to a tighter network with more restricted mobility, which led to materials with higher stiffness and hardness that restricted their stress–strain characteristics.

To sum up, in the present study our research group have proved that the triglycidyl derivative of eugenol, 3EPOEU, is a safe and environmentally friendly alternative to DGEBA. 3EPOEU leads to materials with good thermal and mechanical properties that can replace conventional epoxy monomers in some technological applications. 

## Figures and Tables

**Table 1:** Weight percentages of epoxy monomers and amines used in the curing formulations.

Formulation	3EPOEU [%]	DGEBA [%]	JEF [%]	IPDA [%]
DG-JEF	0	62.9	37.1	0
3EPOEU-JEF	47.5	0	52.5	0
DG-IPDA	0	81.0	0	19.0
3EPOEU-IPDA	69.6	0	0	30.4

**Table 2:** Calorimetric data of the curing of the formulations studied.

Sample	*T*_peak_ [°C] ^1^	Δ*H* [J/g] ^2^	Δ*H* [kJ/mol ee] ^3^	*T*_g_ [°C] ^4^
DG-JEF	139	395	114	50
3EPOEU-JEF	156	428	88	59
DG-IPDA	130	509	114	124
3EPOEU-IPDA	130	655	92	174

^1^ Temperature of the maximum of the exotherm; ^2^ Enthalpy released during curing by gram; ^3^ Enthalpy released during curing by mol of epoxy equivalent; ^4^ Glass transition temperature of the final cured material.

**Table 3:** Thermogravimetric data of the formulations studied.

Sample	*T*_5%_ [°C] ^a^	*T*_max_ [°C] ^b^	Char Residue [%]
DG-JEF	354	381	7.6
3EPOEU-JEF	314	365	15.4
DG-IPDA	348	373	9.6
3EPOEU-IPDA	317	339	23.5

^a^ Temperature of 5% of weight loss in N_2_ atmosphere; ^b^ Temperature of the maximum rate of degradation in N_2_ atmosphere.

**Table 4:** Thermomechanical data of the materials studied.

Sample	*T*_tanδ_ [°C] ^1^	tan δ Peak ^2^	FWHM [°C] ^3^	*E′*_r_ [MPa] ^4^
DG-JEF	62	1.6	9	12
3EPOEU-JEF	73	0.7	17	35
DG-IPDA	174	0.8	12	45
3EPOEU-IPDA	204	0.2	66	214

^1^ Temperature of the maximum of tanδ peak; ^2^ Height of the tanδ peak; ^3^ Width at half height of the tanδ peak; ^4^ Storage modulus at T of the tanδ peak + 40 °C in the rubbery state.

**Table 5:** Mechanical data of the materials studied.

Sample	*E* [MPa] ^1^	σ_b_ [MPa] ^2^	ε_b_ [%] ^3^	Microindentation Hardness [HV]
DG-JEF	1890	30.0	10.7	8.8 ± 0.3
3EPOEU-JEF	1853	22.8	5.7	9.4 ± 0.3
DG-IPDA	3096	73.6	5.9	12.5 ± 0.5
3EPOEU-IPDA	6298	53.5	2.1	13.9 ± 0.4

^1^ Young’s Modulus determined under flexural conditions; ^2^ Stress at break; ^3^ Strain at break.
